# Community engagement, social context and coverage of mass anti-malarial administration: Comparative findings from multi-site research in the Greater Mekong sub-Region

**DOI:** 10.1371/journal.pone.0214280

**Published:** 2019-03-25

**Authors:** Christopher L. Pell, Bipin Adhikari, May Myo Thwin, Ladda Kajeechiwa, Suphak Nosten, Francois H. Nosten, Kate M. Sahan, Frank M. Smithuis, Thuy-Nhien Nguyen, Tran Tinh Hien, Rupam Tripura, Thomas J. Peto, Nou Sanann, Chea Nguon, Tiengkham Pongvongsa, Koukeo Phommasone, Mayfong Mayxay, Mavuto Mukaka, Pimnara Peerawaranun, Nils Kaehler, Phaik Yeong Cheah, Nicholas P. J. Day, Nicholas J. White, Arjen M. Dondorp, Lorenz von Seidlein

**Affiliations:** 1 Amsterdam Institute for Global Health and Development (AIGHD), Amsterdam, The Netherlands; 2 Centre for Social Science and Global Health, University of Amsterdam, Amsterdam, The Netherlands; 3 Mahidol Oxford Tropical Medicine Research Unit, Faculty of Tropical Medicine, Mahidol University, Bangkok, Thailand; 4 Centre for Tropical Medicine and Global Health, Nuffield Department of Medicine, University of Oxford, Oxford, United Kingdom; 5 Shoklo Malaria Research Unit, Mahidol-Oxford Tropical Medicine Research Unit, Faculty of Tropical Medicine, Mahidol University, Mae Sot, Thailand; 6 Sorbonne Universités, UPMC Univ Paris 06, UPMC UMRS CR7, Paris, France; 7 Ethox Centre and Wellcome Centre for Ethics and Humanities, Nuffield Department of Population Health, Big Data Institute, Li Ka Shing Centre for Health Information and Discovery, University of Oxford, Oxford, United Kingdom; 8 Medical Action Myanmar, Yangon, Myanmar; 9 Myanmar Oxford Clinical Research Unit, Yangon, Myanmar; 10 Oxford University Clinical Research Unit, Wellcome Trust Asia Programme, Ho Chi Minh City, Vietnam; 11 National Center for Parasitology, Entomology and Malaria Control, Phnom Penh, Cambodia; 12 Savannakhet Provincial Health Department, Savannakhet Province, Laos; 13 Lao-Oxford-Mahosot Hospital-Wellcome Trust Research Unit (LOMWRU), Microbiology Laboratory, Vientiane, Lao PDR; 14 Institute of Research and Educational Development, University of Health Sciences, Vientiane, Lao PDR; Makerere University, UGANDA

## Abstract

**Background:**

Between 2013 and 2017, targeted malaria elimination (TME), a package of interventions that includes mass drug administration (MDA)–was piloted in communities with reservoirs of asymptomatic *P*. *falciparum* across the Greater Mekong sub-Region (GMS). Coverage in target communities is a key determinant of the effectiveness of MDA. Drawing on mixed methods research conducted alongside TME pilot studies, this article examines the impact of the community engagement, local social context and study design on MDA coverage.

**Methods and findings:**

Qualitative and quantitative data were collected using questionnaire-based surveys, semi-structured and in-depth interviews, focus group discussions, informal conversations, and observations of study activities. Over 1500 respondents were interviewed in Myanmar, Vietnam, Cambodia and Laos. Interview topics included attitudes to malaria and experiences of MDA. Overall coverage of mass anti-malarial administration was high, particularly participation in at least a single round (85%). Familiarity with and concern about malaria prompted participation in MDA; as did awareness of MDA and familiarity with the aim of eliminating malaria. Fear of adverse events and blood draws discouraged people. Hence, community engagement activities sought to address these concerns but their impact was mediated by the trust relationships that study staff could engender in communities. In contexts of weak healthcare infrastructure and (cash) poverty, communities valued the study’s ancillary care and the financial compensation. However, coverage did not necessarily decrease in the absence of cash compensation. Community dynamics, affected by politics, village conformity, and household decision-making also affected coverage.

**Conclusions:**

The experimental nature of TME presented particular challenges to achieving high coverage. Nonetheless, the findings reflect those from studies of MDA under implementation conditions and offer useful guidance for potential regional roll-out of MDA: it is key to understand target communities and provide appropriate information in tailored ways, using community engagement that engenders trust.

## Introduction

Given the devastating consequences of previous spread of drug-resistant malaria from the Greater Mekong Sub-Region (GMS) to sub-Saharan Africa, there is increasing concern about the potential spread of artemisinin-resistant *Plasmodium falciparum* [1–5].Because the global spread of artemisinin resistance could reverse the substantial gains in malaria control achieved in recent years the malaria research community has redoubled efforts to evaluate *P*. *falciparum* elimination strategies in the GMS.

Mass antimalarial drug administration (MDA) has been proposed as a way to eliminate *P*. *falciparum* infections rapidly from transmission foci [6, 7]. This approach aims to interrupt local transmission by offering an artemisinin combination therapy (ACT) to all members of a target population, regardless of malaria infection [6]. The objective is to eliminate asymptomatic carriage of malaria parasites and provide a period of chemoprophylaxis which prevents reinfection. Targeted malaria elimination (TME)–a package of interventions that includes MDA and the strengthening of village malaria worker networks–has been recently piloted in communities with reservoirs of asymptomatic *P*. *falciparum* across the GMS: Myanmar [8–13], Cambodia [14–17], Vietnam [18, 19] and Laos [20–24].

The success of MDA is predicated on the characteristics of the antimalarial regimen, the local dynamics of malaria transmission and coverage in the target communities [25]. Mathematical models suggest that to interrupt local malaria transmission, coverage in target populations must exceed 80% [25, 26]. Achieving this level of uptake in communities is challenging for several reasons: delivering a multi-day antimalarial regimen to all the members of a community requires considerable human and logistical resources; the areas where malaria transmission persists in the GMS are often isolated and home to poor and mobile communities; and in an era of decreasing malaria incidence, populations might question the benefits of taking anti-malarial drugs when seemingly healthy [8–16, 18–23, 27, 28].

Past malaria-related MDAs have often incorporated efforts to encourage the members of target groups to participate, yet such attempts have been often poorly documented and recorded mixed success [25]. All the TME pilot studies incorporated a community engagement strategy that sought to promote coverage of the intervention package in target populations. Although a common term in global health research, community engagement has varied connotations: some researchers, as in TME, prioritize its *instrumental* contribution to the success of studies, in terms of achieving specific research objectives or health outcomes; others focus on its *intrinsic* value for ethical research practice [25, 29–31]. Often the impact of community engagement on study objectives is complicated by external factors–elements of the local context [21]. For example, in the case of indoor residual spraying for malaria control in Mozambique, despite the efforts of study staff to promote uptake, political divisions strongly influenced participation [32].

Drawing on a programme of mixed methods research that was conducted alongside TME pilot studies across the GMS [9, 12, 14, 16, 18, 21–23], this article examines the impact of the community engagement, local social context and study design on the coverage of MDA. With a view to informing the design of future MDAs across the region, the various contributions of these factors are analysed comparatively. A comparative approach facilitates the generation of key insights from a variety of contexts that are relevant for potential implementation across a diverse region. This approach also allows the identification and interrogation of issues that might been taken for granted had data collection focused on a single site. It is particularly useful as a means to draw broader lessons from the often site-specific entanglement of community engagement and local social circumstances.

## Methods

### Ethics approval and consent to participate

In Cambodia, approval was obtained from the National Ethics Committee for Health Research Cambodia (NECHR 0042& 0051), the Oxford Tropical Research Ethics Committee (OXTREC; 1017–13), and the study was registered on clinicaltrials.gov (NCT01872702). Written informed consent was obtained from all TME study participants. Verbal consent was obtained prior to interviews and this was audio recorded. Verbal rather than written consent was obtained because participation posed minimal risk to the respondents. In Laos, ethical approval for the study was received from the Lao National Ethics Committee for Health Research (Ref. No. 013-2015/NECHR), Government of the Lao PDR and the Oxford Tropical Research Ethics Committee (1015–13). Written informed consent was sought from each participant before each interview. In Vietnam, ethical approval was received from the Institute of Malariology, Parasitology and Entomology in Ho Chi Minh City (185/HDDD), the Institute of Malariology, Parasitology and Entomology in Qui Nhon and the Oxford Tropical Research Ethics Committee (1015‑13). Written informed consent was obtained from each participant.

In Myanmar, the studies were approved by the Ethics Review Committee of the Department of Medical Research (Ref: 74/Ethics 2014) and the Oxford Tropical Research Ethics Committee (23–15; 1015–13), the Tak Province Community Ethics Advisory Board and the village committees. Participants gave written informed consent. The TMT study, Myanmar II was registered with clinicaltrials.gov (id: NCT01872702).

### Settings

**Fig 1:**
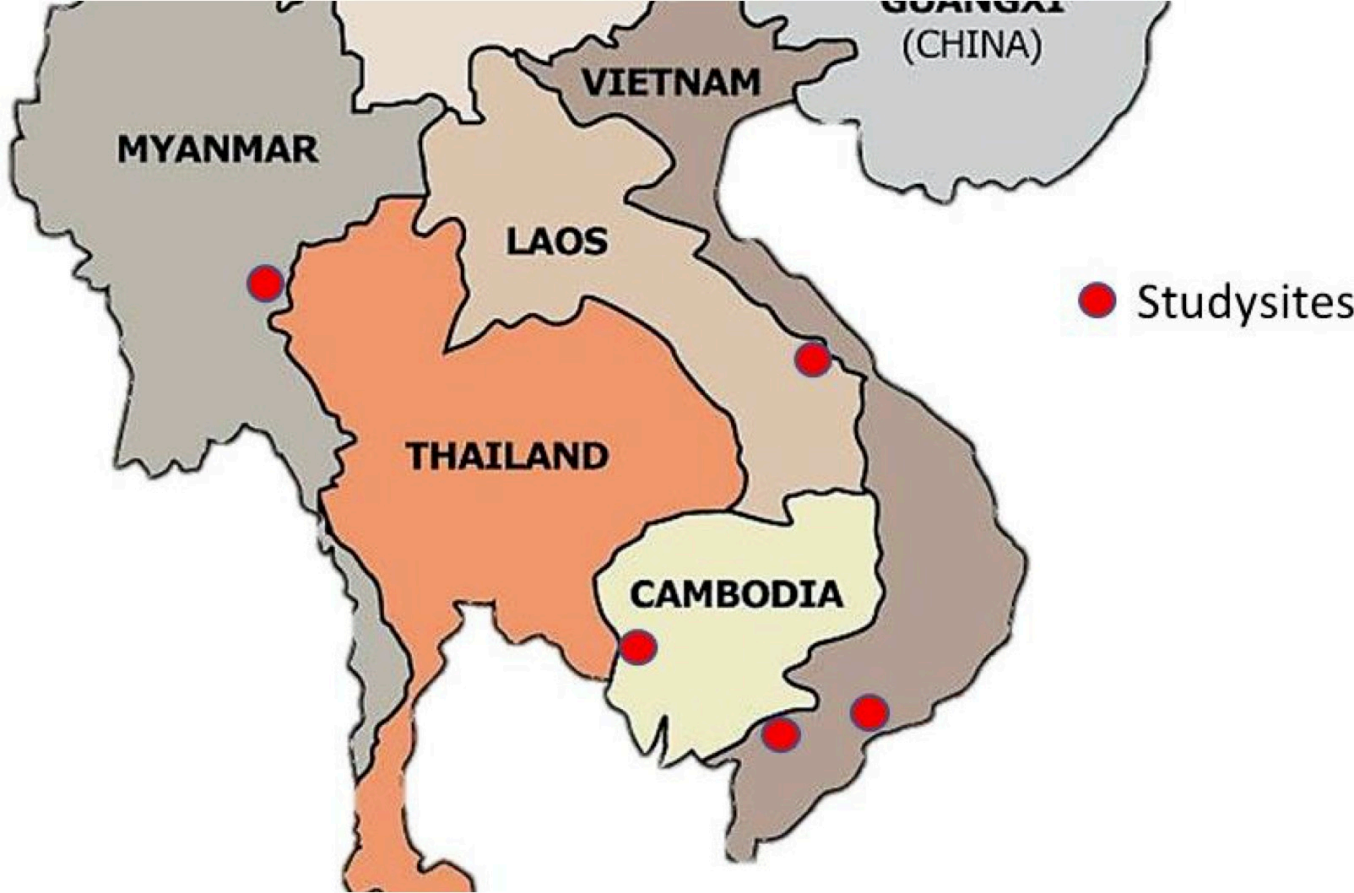
Targeted malaria elimination study sites in Myanmar, Cambodia, Laos and Vietnam.

The following section provides an overview of the studies sites (Fig 1 indicates locations). Full details are available in the site-specific articles. Table 1 summarizes the prevalence of *Plasmodium* infections prior to TME, and highlights the size of the asymptomatic reservoir revealed by ultra-sensitive molecular methods.

**Table 1:** Malaria prevalence at TME sites based on surveys prior to MDA (all species).

	Sites								Overall
	Myanmar [33]		Vietnam [33]		Cambodia [33]		Laos [20]		
*Plasmodium* prevalence	Total	Positive	Total	Positive	Total	Positive	Total	Positive	Total (%)
RDT	1384	158 (11%)	2177	65 (3%)	1447	1 (0.1%)	888	18 (2%)	242/5896 (4%)
Microscopy	1532	144 (9%)	2132	77 (4%)	1447	8 (1%)	NA*	NA*	229/5111 (5%)
uPCR	1536	520 (34%)	1992	239 (12%)	1447	229 (16%)	888	175 (20%)	1163/5863 (20%)

*Microscopy were not conducted in Laos TME.

#### Kayin State, Myanmar

In Kayin State, eastern Myanmar, data were collected alongside two studies involving mass anti-malarial administration in separate townships [8, 12]. The Myanmar I study was conducted in four villages located within 10 km of the Thailand-Myanmar border and the Myanmar II study was undertaken in 10 villages in Kyaingseikgyi, the most southern township of Kayin State. Although there were no ongoing conflicts at the time of the studies, in this region, clashes between government armed forces and state-level armed groups have left lasting community divisions and the political situation is unstable. Migration for safety and economic reasons is common. Infrastructure in the area is limited, with villages often accessible only by motorbike or on foot, or not at all in the rainy season, and health facilities few and far between [34]. Access and mobility is also complicated by floods, road blocks and mountainous terrain. The target villagers are home to ethnic groups, including the Burman, Pow Karen, and Sgaw Karen.

#### Binh Phuoc and Ninh Thuan Provinces, Vietnam

In Vietnam, TME was conducted in Dak O Commune, Binh Phuoc Province and in Phuoc ha Commune, Ninh Thuan Province. 1) Binh Phuoc Province is located in the southeast region of the country north-west from Ho Chi Minh City and shares a border with Cambodia. The province has hilly regions with the majority used for perennial cash crops. Bin Phuoc is a rural province inhabited mostly by Vietnamese, it is also home for minorities such as *S’tieng*, *Nung*, *Tay* and *Khmer*. 2) Ninh Thuan Province is located in the south- central coast with high mountains on the western border and near the coast. Ninh Thuan Province is one of the most forested provinces of Vietnam (56%). The major ethnic group is the Kinh, but the province is also populated by the Cham and Raglai ethnic groups.

#### Battambang Province, Cambodia

In Battambang Province, TME was conducted in Samlout District, an area where falciparum malaria has declined over the past fifteen years and malaria transmission is unstable [35]. In this border region, clinical and asymptomatic *P*. *falciparum* infections are associated with travel to local forests and most transmission is thought to occur outside villages [36, 37]. At the time of the study, a programme of activities aimed at understanding and containing artemisinin-resistance had been underway since 2007 [38].

#### Savannakhet Province, Laos

In Laos, TME took place in Nong District, southern Savannakhet Province. This area borders Vietnam and is one of the poorest districts in Laos [3, 39, 40]. The local population is made up of members of Laotian minority groups, who speak a language distinct from the commonly spoken *Pasha Lao* [21]. The villages are comprised of between 300 and 500 residents and are accessible by road [23].

### The TME pilot studies

The impact of antimalarial MDAs was evaluated in a cluster randomised trial, with intervention and control villages. The TME studies were carried-out in collaboration with the local malaria control programmes of Cambodia, Myanmar, Laos and Vietnam, local health authorities and local research institutes.

The unit of randomisation and statistical inference was the village. At each site, early (year 1) versus deferred (year 2) MDA was allocated by restricted randomization within two pairs of villages matched for geographical proximity and parasite prevalence Study villages were selected on the basis of prevalence of asymptomatic *P*. *falciparum* infections detected in earlier prevalence surveys, their size and accessibility [11, 15, 19, 20]. The selected villages across GMS recorded populations of between 239 and 1112.

At all TME sites, MDA followed a similar design. Intervention villages received MDA of dihydroartemisinin piperaquine (DHA-PPQ) for three days and a single low dose primaquine every month for three months. In the control villages, the MDAs were deferred by nine to 12 months. Blood surveys were conducted in intervention and control villages at baseline (M0) and then in quarterly intervals. These surveys involved collection of venous blood sample (3ml from adults and 0.5ml from children ≤ 5years) from all participants. During the study period, study staff visited village malaria workers weekly, to provide diagnostic tests, antimalarials, and collect treatment records [8–12, 14–16, 19–23, 41].

In terms of overall coverage in the target populations, there were notable differences across and within sites and over the three-rounds of MDA (Table 2).

**Table 2:** Coverage of MDA at the TME sties.

TME Site	Completed at least 1 round		Completed at least 2 rounds		Completed 3 rounds		Total target population
	n	%	n	%	n	%	n
**Myanmar I**	1,866	71	1,342	51	911	35	2,634
**Myanmar II**	4,156	90	3,614	78	2,897	63	4,622
**Vietnam**	2,572	94	2,127	78	1,485	54	2,731
**Cambodia**	1,415	85	1,137	68	862	52	1,665
**Laos**	1,526	79	1,446	75	1,362	70	1,935
**Total**	11,535	85	9,666	71	7,517	55	13,587

Residents were categorized as did not participate at all, did not complete a single round (three doses), completed only one round, completed only two rounds, or completed all three rounds. For the estimation of the MDA coverage, the numerator was defined as the number of participants by MDA rounds. The denominator was defined as the *de facto* population at the time of the MDA.

### Community engagement for TME

At each pilot study site, to increase the coverage of mass antimalarial administration, intensive programmes of community engagement were conducted. These activities began from the moment of site selection, generally beginning with discussions about the planned study with people in positions of provincial, district and village leadership.

The programme of community engagement activities was adapted to the specific characteristics of each study site and specific community engagement activities were tailored to local preferences and customs. The design of community engagement was also responsive to events and feedback during the pilot studies. To aid this process, in each study site, a committee was formed of village leaders, village malaria workers, and community volunteers (who were paid a stipend). The committees assisted the study team in designing and implementing community engagement (and other aspects of the pilot studies). Preliminary findings from the programme of qualitative research were also used to inform the ongoing process of tailoring community engagement.

In the target communities, key elements of the engagement activities involved informing potential participants about the purpose of and need for participation of all community members. Information was provided through posters, radio advertisement, and workshops with authorities and local decision makers. Meetings were organized at different scales: informal discussions with individuals or with all household members, whole village assemblies, or meetings with specific population groups (e.g. mothers of young children). In addition, there were activities focusing on children and young people, such as music festivals and theatre in Cambodia.

At all sites, the study teams provided incentives for participation, which were adapted according to the preferences of the target population and the regulatory authorities. Individual cash or non-cash incentives combined with gifts, and/or lotteries were preferred in some sites whereas in others individual incentives were considered inappropriate and community incentives e.g. improved water supply for the entire village were provided. Ancillary care was provided by the study team to all community members. Health education on topics unrelated to the pilot studies, such as family planning, nutrition and vaccinations was provided to community members at their request. For all community members, the study teams provided uninterrupted access to early diagnosis, and adequate treatment of malaria and to insecticide-treated bed nets.

### Data collection

Qualitative data were collected before, during and after the MDAs. To increase the reliability of findings, a range of qualitative data collection techniques were used with various respondent types (Table 3). This enabled triangulation and lessened the potential bias of one particular data collection tool or respondent type. Conducting extended fieldwork in the communities, which included informal conversations with villagers, also enabled the field staff to observe villagers’ behaviours towards TME.

**Table 3:** Data collection methods across various TME sites in the GMS.

Respondents / focus	Data collection activities	Sites							
		Myanmar		Vietnam	Cambodia		Laos		Total
		I	II		2015	2016	2015	2016	
**Community members**	Questionnaire-based survey*	384	0	138	123	240	281	158	1324
	Semi-structured interviews	0	28	0	35	16	0	31	110
	Focus group discussions	0	0	0	4	4	12	0	20
	Informal interviews	0	0	0	0	0	0	88	88
**Community leaders**	In-depth interviews	0	3	0	10	9	0	0	22
**Trial staff**		0	13	0	5	3	6	0	27
**Study activities**	Observations	No	Yes	No	Yes	No	Yes	Yes	
	**Total**								1591

*Only post-MDA and matched data sets from these surveys were included in the analysis

The methods (Table 3) included semi-structured and in-depth (individual) interviews, focus group discussions. The qualitative data were collected by trained field workers, fluent in the local language and English (except in Laos where locals acted as translators for the Laotian and non-Laotian social scientists), who were resident in the field site for between four months and a year.

#### Semi-structured interviews with community members

At each site, trained social scientists conducted semi-structured interviews (SSIs) with respondents from the target villages. In Cambodia and Laos, they were selected at random from a list of adult residents recorded by a household census conducted immediately prior to the study. A random sample of study participants (men or women over the age of 18) was chosen because TME is a community-wide approach and this enabled the research team to collect an overall impression of the community response to the intervention. Respondents were interviewed around seven days after rounds one, two and three of the mass anti-malarial administrations. The first interview was audio-recorded, transcribed and translated. Detailed notes were taken during the second and third interviews. In Myanmar, respondents were selected based on a mixture of snowballing, diversity, and pragmatic sampling approaches, which were dependent on access to field sites. Individual interviews with community members took place at the respondent’s home or in the immediate vicinity.

#### In-depth interviews with study staff

In Cambodia, Laos and Myanmar, trained social scientists conducted in-depth interviews (IDIs) with TME staff members involved to inform the design and implementation of TME and community engagement. Later at various points during and after MDA, community members who refused to participate or who did not complete all three doses (in all three rounds) were also interviewed: in Cambodia, respondents were selected randomly from study records and interviewed; in Myanmar, interviewed community members included partial MDA participants, who were contacted through snow-ball sampling. Interviews with study staff took place at convenient locations, such as a room in the site or head office.

#### Focus group discussions

Focus groups were conducted by trained social scientists in Cambodia and Laos. In Cambodia, FGD participants were purposefully selected by the village leaders, who acted as gatekeepers. In Laos, FGD participants (men or women over the age of 18) were selected at random (by lot) from the large number of community members who expressed a desire to be interviewed at village meetings. In Cambodia FGDs were held in the compounds of the VMWs, and in the residence of one of the participants, in Laos.

#### Interview topics

Interview topics included experience and understanding of malaria, anti-malarial drugs and the TME project (including the community engagement activities). Focus groups also explored issues of the movement of forest-goers. Interviews covered the challenges associated with undertaking the project and the community engagement activities, including possible strategies to counteract those challenges.

#### Observations

During the project, the researchers recorded their observations of study activities and community engagement. In Cambodia, this included, specifically, taking notes based on meetings with village leaders that were conducted during the design phase of the community engagement approach. In Laos, this included three field staff, taking notes with relevant reflection on all the activities in the villages. A total of 130 field notes were taken.

### Qualitative data processing and analysis

The SSIs, IDIs and FGDs, were audio recorded and transcribed verbatim and translated into English. For quality control a sample of transcripts were checked by a second translator, who compared them with the original audio recording. Observations of study activities and relevant events in the community during the study periods were recorded as hand-written field notes (in either the local language or English, depending on the preference of the field staff) and subsequently typed up (and translated if necessary) in English. At each site, there was extensive debriefing of study staff, which entailed the discussion of the emerging themes in observations and interviews.

At each site, a qualitative data analysis software package (QSR NVivo 10) was used to develop a codebook and conduct line-by-line coding. Data analysis combined inductive and deductive elements: analytical categories were developed from the initial research questions and emerged during the analysis process. This meant that some elements of the codebooks were homogenous across the sites (those based on the same initial questions) but heterogeneity was introduced through the use of inductive codes. At each site, line-by-line coding of data was conducted by or in consultation with the first author. For the comparative analysis across the sites, a combined NVivo 11 project was created. Using this merged project, analysis continued by exploring the patterns of the codes across the transcripts/data sources and sites, identifying exceptional cases and examining differences in the coded text. The results of preliminary analyses were discussed with staff from all sites. The content of the codes was used to develop the themes presented in the results

### The questionnaire-based survey data

A questionnaire consisting of questions about socio-demographic characteristics, knowledge attitude, perceptions of malaria and MDA was administered to residents of TME villages in Cambodia, Myanmar (I), Laos and Vietnam. The questionnaire was adapted from previous research conducted in The Gambia [42]. In each site, the questionnaire was adapted according to site-specific characteristics, such as local ethnicities, religious groups and occupations [9, 18, 22, 41]. Questionnaire data were collected after obtaining written informed consent, by face-to-face interview with an adult (above 18 years) from each household in the study villages after the completion of the MDA.

All collected data from the TME sites were examined for common questions and matching variables. Matched data sets from each site were appended for a final data set. Initial analysis included exploration of the association between socio-demographic variables, including knowledge and perceptions with the complete participation in MDA (none, incomplete and complete) using either Chi-squared test or Fisher’s exact test where appropriate. Based on previous research, initial participation in MDA were classified into three categories “none” meant no participation at all, “incomplete” meant participation in fewer than nine doses and “complete” meant participation in all three rounds (nine doses) [9, 18, 22].

Logistic regression models were used to test the association between predisposing variables and outcome variables (participation in MDA). All variables significant in the univariate analysis or of thematic relevance (for example, knowledge on malaria, experience of MDA and perceptions on MDA), were included in the final model. Complete participation in MDA was coded as 1 compared to none and incomplete (which were coded as 0) and underwent univariate and multivariate analysis for odds ratios. Statistical analyses were performed using Stata 14.1 (StataCorp LP, College Station, TX, USA).

## Results

### Coverage of mass antimalarial administration

Across the TME study sites, coverage of mass anti-malarial administration was high, particularly participation in at least a single round (85%; Table 2). Of the 840 respondents who participated in the questionnaire-based survey from the four TME sites, 462 (55%) completed all three rounds of MDA, 225 (27%) completed one or two rounds, and 153 (18%), did not take part at all. The majority took part in at least one round of MDA (687/840; 82%) **(Table 4).** As reflected in the overall coverage, there were notable differences in coverage across (and within) sites and during the three-rounds of MDA.

**Table 4:** Socio-demographic characteristics of survey respondents in relation to MDA (n = 840).

Characteristics	Participation in MDA			Total (n = 840)	p-value*
	None (n = 153)	Incomplete (n = 225)	Complete (n = 462)		
	Number (%)	Number (%)	Number (%)	Number (%)	
**Countries (n = 840)**					
Cambodia	19 (12.4)	63 (28)	81 (17.5)	163 (19.4)	**<0.001**
Laos	8 (5.2)	9 (4)	141 (30.5)	158 (18.8)	
Myanmar I	80 (52.3)	133 (59.1)	171 (37)	384 (45.7)	
Vietnam	46 (30.1)	20 (8.9)	69 (14.9)	135 (16.1)	
**Age group (n = 840)**					
≤ 31 years	50 (32.7)	75 (33.3)	157 (34)	282 (33.6)	0.79
32 to 46 years	49 (32)	75 (33.3)	165 (35.7)	289 (34.4)	
≥ 47 years	54 (35.3)	75 (33.3)	140 (30.3)	269 (32)	
Mean = 39.4±13.3					
**Sex (n = 840)**					
Female	87 (56.9)	151 (67.1)	230 (49.8)	468 (55.7)	**<0.001**
Male	66 (43.1)	74 (32.9)	232 (50.2)	372 (44.3)	
**Religion (n = 840)**					
Ancestral worship	2 (1.3)	3 (1.3)	3 (0.6)	8 (1)	**<0.001**
Animist	8 (5.2)	8 (3.6)	138 (29.9)	154 (18.3)	
Atheist	35 (22.9)	5 (2.2)	51 (11)	91 (10.8)	
Buddhist	98 (64.1)	193 (85.8)	248 (53.7)	539 (64.2)	
Christian	9 (5.9)	12 (5.3)	21 (4.5)	42 (5)	
Other	1 (0.7)	4 (1.8)	1 (0.2)	6 (0.7)	
**Marital status (n = 826)**					
In relationship	134 (89.9)	205 (94)	424 (92.4)	763 (92.4)	0.34
Not in relationship	15 (10.1)	13 (6)	35 (7.6)	63 (7.6)	
**Literacy (n = 840)**					
Illiterate	67 (43.8)	79 (35.1)	221 (47.8)	367 (43.7)	**0.007**
Literate	86 (56.2)	146 (64.9)	241 (52.2)	473 (56.3)	
**Occupation (n = 812)**					
Farmer	134 (89.3)	206 (92)	342 (78.1)	682 (84)	**<0.001**
Non-farmer	16 (10.7)	18 (8)	96 (21.9)	130 (16)	
**Monthly income (n = 319)**					
Lower income	16 (59.3)	40 (55.6)	181 (82.3)	237 (74.3)	**<0.001**
Higher income	11 (40.7)	32 (44.4)	39 (17.7)	82 (25.7)	
**Migration (n = 840)**					
Native	81 (53.3)	95 (42.2)	244 (52.8)	420 (50.1)	**0.02**
Migrated	71 (46.7)	130 (57.8)	218 (47.2)	419 (49.9)	

*Fisher exact test and Chi-squared test

The coverage of mass antimalarial administration was influenced by a complex set of factors, with community engagement and study design intertwined with the local study contexts. The following sections outline the key issues that influenced uptake of MDA among members of the target communities across the five sites.

### Malaria as a health concern

**Fig 2:**
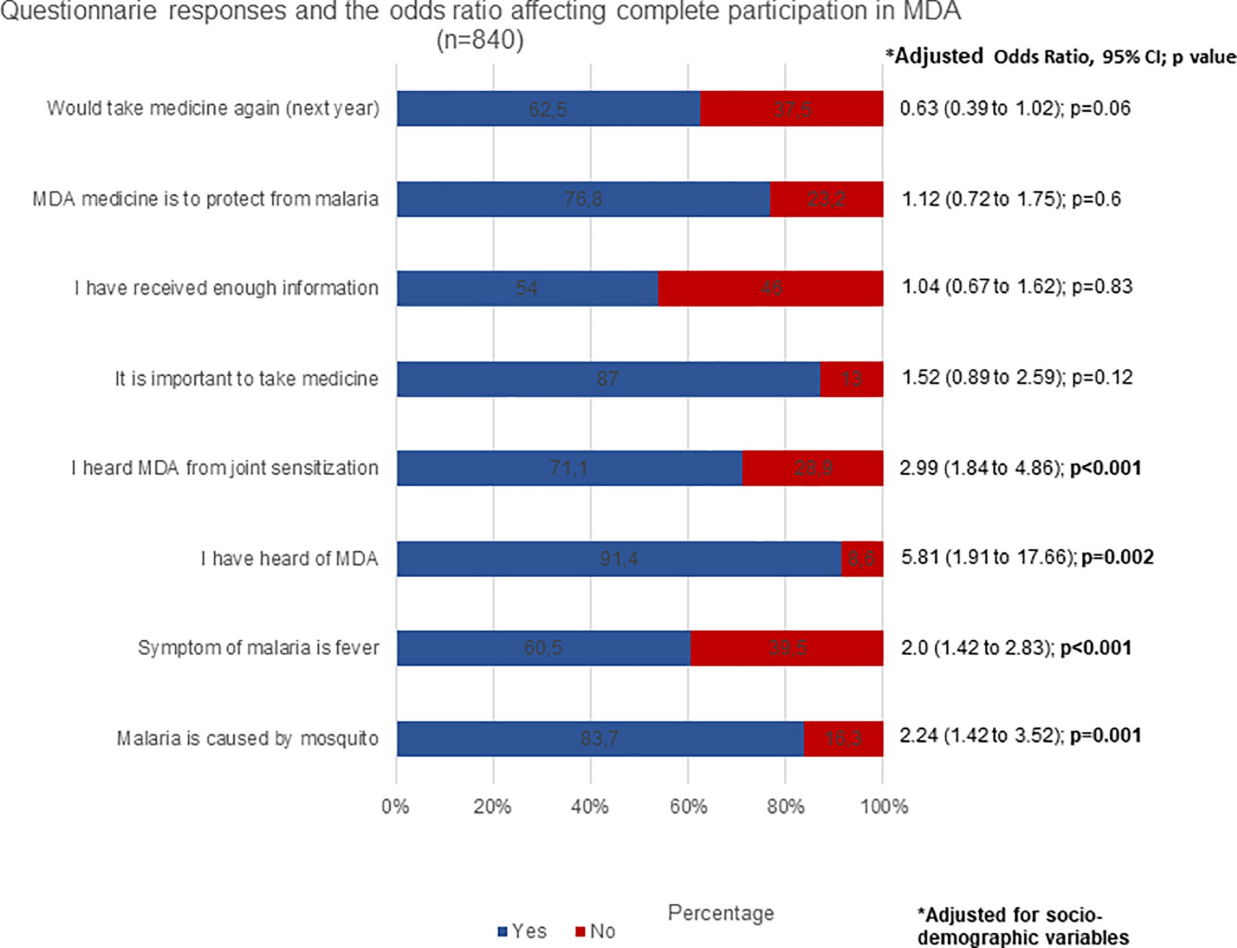
Factors independently associated with completing all MDA rounds.

Across the sites, questionnaire respondents who were familiar with what transmits malaria (malaria is transmitted by mosquitoes: 414/462; 89.6%; p<0.001), symptoms such as fever (313/462; 67.7%; p<0.001) and shivering (319/462; 69.3%; p = 0.02), were more likely to complete participation in all three rounds of MDA **(Table 5)**. A multivariate analysis showed that knowing what causes malaria and its symptoms was associated with significantly higher odds of completing the participation in all three rounds of MDA **(Fig 2 and S1 Table)**.

**Table 5:** Knowledge about malaria in relation to participation in MDA rounds (n = 840).

Characteristics	Participation in MDA			Total (n = 840)	p-value*
	None (n = 153)	Incomplete (n = 225)	Complete (n = 462)		
	Number (%)	Number (%)	Number (%)	Number (%)	
**Cause of malaria**					
**Mosquitoes (n = 840)**					
Yes	116 (75.8)	173 (76.9)	414 (89.6)	703 (83.7)	**<0.001**
No	37 (24.2)	52 (23.1)	48 (10.4)	137 (16.3)	
**Eating certain food (n = 519)**					
Yes	4 (3.2)	7 (4.6)	7 (2.9)	18 (3.5)	0.66
No	122 (96.8)	146 (95.4)	233 (97.1)	501 (96.5)	
**Rain (n = 519)**					
Yes	2 (1.6)	3 (2)	1 (0.4)	6 (1.2)	0.33
No	124 (98.4)	150 (98)	239 (99.6)	513 (98.8)	
**Unhygienic surrounding (n = 682)**					
Yes	5 (3.4)	8 (3.7)	17 (5.3)	30 (4.4)	0.55
No	140 (96.6)	208 (96.3)	304 (94.7)	652 (95.6)	
**Water (n = 682)**					
Yes	10 (6.9)	24 (11.1)	33 (10.3)	67 (9.8)	0.39
No	135 (93.1)	192 (88.9)	288 (89.7)	615 (90.2)	
**Forest (n = 321)**					
Yes	1 (3.7)	5 (6.9)	6 (2.7)	12 (3.7)	0.25
No	26 (96.3)	67 (93.1)	216 (97.3)	309 (96.3)	
**Germ (n = 163)**					
Yes	0 (0)	2 (3.2)	2 (2.5)	4 (2.5)	0.73
No	19 (100)	61 (96.8)	79 (97.5)	159 (97.5)	
**Soil (n = 163)**					
Yes	0	4 (6.3)	4 (4.9)	8 (4.9)	0.53
No	19 (100)	59 (93.7)	77 (95.1)	155 (95.1)	
**Don't know (n = 677)**					
Yes	27 (20.1)	35 (21.6)	25 (6.6)	87 (12.9)	**<0.001**
No	107 (79.9)	127 (78.4)	356 (93.4)	590 (87.1)	
**Symptoms of malaria**					
**Fever (n = 838)**					
Yes	68 (44.4)	126 (56.5)	313 (67.7)	507 (60.5)	**<0.001**
No	85 (55.6)	97 (43.5)	149 (32.3)	331 (39.5)	
**Headache (n = 831)**					
Yes	92 (60.5)	136 (61.8)	305 (66.4)	533 (64.1)	0.29
No	60 (39.5)	84 (38.2)	154 (33.6)	298 (35.9)	
**Shivering (n = 831)**					
Yes	89 (58.9)	136 (61.8)	319 (69.3)	544 (65.5)	**0.02**
No	62 (41.1)	84 (38.2)	141 (30.7)	287 (34.5)	
**Vomiting (n = 829)**					
Yes	64 (42.7)	107 (48.4)	159 (34.7)	330 (39.8)	**0.002**
No	86 (57.3)	114 (51.6)	299 (65.3)	499 (60.2)	
**Diarrhea (n = 454)**					
Yes	1 (1.4)	3 (3.3)	12 (4.1)	16 (3.5)	0.51
No	72 (98.6)	87 (96.7)	279 (95.9)	438 (96.5)	
**Jaundice (n = 161)**					
Yes	0 (0)	2 (3.3)	3 (3.7)	5 (3.1)	0.7
No	19 (100)	59 (96.7)	78 (96.3)	156 (96.9)	
**Body pain (n = 454)**					
Yes	9 (12.3)	27 (30)	59 (20.3)	95 (20.9)	**0.02**
No	64 (87.7)	63 (70)	232 (79.7)	359 (79.1)	
**Sore throat (n = 161)**					
Yes	0 (0)	0 (0)	1 (1.2)	1 (0.6)	0.6
No	19 (100)	61 (100)	80 (98.8)	160 (99.4)	
**Dizziness (n = 135)**					
Yes	8 (17.4)	2 (10))	10 (14.5)	20 (14.8)	0.73
No	38 (82.6)	18 (90)	59 (85.5)	115 (85.2)	
**Don't know (n = 297)**					
Yes	17 (30.9)	5 (16.7)	26 (12.3)	48 (16.2)	**0.004**
No	38 (69.1)	25 (83.3)	186 (87.7)	249 (83.8)	

*Fisher exact test and Chi-squared test

At all sites, community members described malaria as a local health concern. Worries about getting infected with malaria were linked to its economic consequences, particularly having to take time off from agricultural work. Personal experiences of malaria infection or experience of close friends and relatives compounded concerns about the disease. In Cambodia, these concerns about malaria persisted despite the decline in malaria incidence since the early 2000s. Respondents’ worries were based on their recollections of disease episodes from before this time, which still remained influential 20 to 30 years later.

Worries about malaria were embedded in a general familiarity with the disease’s symptoms but understanding of malaria transmission varied. In Cambodia–a site that has seen, in the past decade, intensive activities to stem the spread of drug resistance–respondents were very aware that mosquito bites were associated with malaria transmission. Elsewhere, other explanations, particularly poor hygiene, were thought to play a prominent role in malaria aetiology. Spending time in the forest was seen as contributing to infection: in Myanmar, malaria was termed “forest sickness” yet the mechanism of malaria transmission was less clear to respondents. In Laos, this ambiguity had the unexpected consequence of making the concept of asymptomatic malaria easier to grasp for community members: respondents were open to the idea of asymptomatic infection among residents, in contrast to outsiders (for example, TME staff) who they viewed as “cleaner”, and who did not visit the forests. For community members, malaria–asymptomatic or symptomatic–was a disease caused by lifestyle and place, and not parasites.

The reported use of preventive measures also varied across the sites. In Cambodia, respondents referred to wearing long-sleeved clothes and using ITNs to prevent mosquito-bites. In Myanmar, the use of ITNs was described as uncommon and influenced negatively by high night-time temperatures that made sleepers more uncomfortable when airflow was attenuated by ITNs. During the 2016 interviews in Cambodia, respondents described anti-malarials, specifically those delivered as part of TME, as a form of malaria prevention or individual malaria “elimination”.

At all the sites, community engagement activities included malaria-related education. This took place in community meetings and more informally during TME staff members’ interactions with residents of the target communities. In Cambodia, drama performances were used as a means of communicating messages about malaria transmission, and prevention and control. These well-received performances involved the participation of community members alongside trained performers. At all sites, visual methods were used to describe the phenomenon of asymptomatic malaria infection and explain how it could lead to symptomatic infections among other community members and contribute to continued transmission.

### Understanding mass antimalarial administration

Among the survey respondents, having knowledge of MDA (heard of MDA: 451/462; 97.6%; p<0.001), hearing it from sources linked to the pilot studies, such as during community meetings(380/462; 83.5%; p<0.001), receiving adequate information about MDA (274/312; 87.8%; p<0.001), responses such as “it is important to take medicine” (416/455; 91.4%; p = 0.001), “the purpose of MDA medicine is to protect from malaria” (318/381;83.5%; p<0.001), and an intention to take part in MDA again (292/310; 94.1%; p<0.001) were each associated with complete participation **(Table 6).** Logistic regression models showed that people hearing about MDA (AOR = 5.81; CI = 1.91 to 17.66; p = 0.002) and hearing about it during community engagement activities (AOR = 2.99; CI = 1.84 to 4.86; p<0.001) were significantly more likely to complete the participation in MDA rounds than people who were not informed in this way **(Fig 2 and S1 Table)**.

**Table 6:** Knowledge, perceptions and experiences related to MDA (n = 840).

Characteristics	Participation in MDA			Total (n = 840)	p-value*
	None (n = 153)	Incomplete (n = 225)	Complete (n = 462)		
	Number (%)	Number (%)	Number (%)	Number (%)	
**Have you heard of MDA (n = 839)**					
Yes	116 (76.3)	201 (89.3)	451 (97.6)	768 (91.5)	**<0.001**
No	28 (18.4)	17 (7.6)	10 (2.2)	55 (6.6)	
Don’t know	8 (5.3)	7 (3.1)	1 (0.2)	16 (1.9)	
**Where did you hear it from**					
**Community engagement (n = 819)**					
Yes	78 (52.3)	124 (57.7)	380 (83.5)	582 (71.1)	**<0.001**
No	71 (47.7)	91 (42.3)	75 (16.5)	237 (28.9)	
**Banners (n = 518)**					
Yes	6 (4.8)	9 (5.9)	30 (12.5)	45 (8.7)	**0.016**
No	119 (95.2)	144 (94.1)	210 (87.5)	473 (91.3)	
**Radio (n = 592)**					
Yes	9 (7)	3 (1.7)	25 (8.7)	37 (6.3)	**0.009**
No	120 (93)	174 (98.3)	261 (91.3)	555 (93.8)	
**Household member (n = 518)**					
Yes	14 (11.2)	11 (7.2)	12 (5)	37 (7.1)	0.09
No	111 (88.8)	142 (92.8)	228 (95)	481 (92.9)	
**Neighbor (n = 750)**					
Yes	20 (14.6)	33 (17.7)	39 (9.1)	92 (12.3)	**0.008**
No	117 (85.4)	153 (82.3)	388 (90.9)	658 (87.7)	
**Village head (n = 301)**					
Yes	6 (25)	7 (11.3)	110 (89.4)	123 (40.9)	**<0.001**
No	18 (75)	55 (88.7)	105 (48.8)	178 (59.1)	
**Villager (n = 518)**					
Yes	2 (1.6)	1 (0.7)	3 (1.3)	6 (1.2)	0.75
No	123 (98.4)	152 (99.3)	237 (98.8)	512 (98.8)	
**Don’t know (n = 676)**					
Yes	19 (14.3)	7 (4.3)	7 (1.8)	33 (4.9)	**<0.001**
No	114 (85.7)	155 (95.7)	374 (98.2)	643 (95.1)	
**It is important to take medicine (n = 815)**					
Yes	123 (85.4)	192 (88.9)	416 (91.4)	731 (89.7)	**0.001**
No	10 (6.9)	5 (2.3)	27 (5.9)	42 (5.2)	
Don’t know	11 (7.6)	19 (8.8)	12 (2.6)	42 (5.2)	
**Received enough information on MDA (n = 582)**					
Yes	77 (64.7)	103 (68.2)	274 (87.8)	454 (78)	**<0.001**
No	18 (15.1)	24 (15.9)	28 (9)	70 (12)	
Don’t know	24 (20.2)	24 (15.9)	10 (3.2)	58 (10)	
**Purpose of the MDA medicine**					
**To kill the malaria parasite (n = 158)**					
Yes	2 (25)	6 (66.7)	132 (93.6)	140 (88.6)	**<0.001**
No	6 (75)	3 (33.3)	9 (6.4)	18 (11.4)	
**To protect from malaria (n = 676)**					
Yes	71 (53.4)	130 (80.2)	318 (83.5)	519 (76.8)	**<0.001**
No	62 (46.6)	32 (19.8)	63 (16.5)	157 (23.2)	
**Mosquitoes will not bite me (n = 518)**					
Yes	0 (0)	2 (1.3)	3 (1.3)	5 (1)	0.44
No	125 (100)	151 (98.7)	237 (98.8)	513 (99)	
**No need to sleep under the mosquito net (n = 518)**					
Yes	0 (0)	1 (0.7)	0 (0)	1 (0.2)	0.3
No	125 (100)	152 (99.3)	240 (100))	517 (99.8)	
**Gives me energy (n = 676)**					
Yes	14 (10.5)	12 (7.4)	13 (3.4)	39 (5.8)	**0.006**
No	119 (89.5)	150 (92.6)	368 (96.6)	637 (94.2)	
**Don’t know**					
Yes	20 (15)	14 (8.6)	14 (3.7)	48 (7.1)	**<0.001**
No	113 (85)	148 (91.4)	367 (96.3)	628 (92.9)	
**Will take MDA medicine next year (n = 591)**					
Yes	97 (75.2))	136 (89.5)	292 (94.2)	525 (88.8)	**<0.001**
No	16 (12.4)	7 (4.6)	5 (1.6)	28 (4.7)	
Don’t know	10 (7.8)	7 (4.6)	5 (1.6)	22 (3.7)	
Conditional	6 (4.7)	2 (1.3)	8 (2.6)	16 (2.7)	
**MDA is important (n = 749)**					
Yes	125 (88.7)	198 (92.1)	372 (94.7)	695 (92.8)	0.13
No	4 (2.8)	3 (1.4)	6 (1.5)	13 (1.7)	
Maybe	1 (0.7)	0 (0)	0 (0)	1 (0.1)	
Don’t know	11 (7.8)	14 (6.5)	15 (3.8))	40 (5.3)	

*Fisher exact test and Chi-squared test

In Cambodia, interview respondents were well aware of TME’s aim of “malaria elimination”. In Laos, “elimination” was mentioned but community members also offered broader descriptions of TME’s contribution to addressing malaria and improving community health. These responses reflected the way in which TME was presented during community engagement activities: during meetings staff reiterated the goal of eliminating malaria and, particularly in Laos, staff appealed to residents to work together to improve their health. Study posters, used extensively in Laos, and other materials emphasized such messages. Community members were also aware of the main TME activities: blood sampling and giving medicines. Opinions of the project, regarding its main aim, malaria elimination, were generally positive: community members viewed malaria as a problem and were content that someone was addressing an issue that had both health and economic consequences. Many community members, and some study staff, were however, less familiar with the details of TME and its rationale.

### Perceptions of adverse events and blood draws

Particularly in Cambodia and Laos, respondents who participated in the MDA attributed a range of complaints to the anti-malarial drugs received. The complaints included vomiting, nausea, shivering, head/stomach ache, dizziness, fatigue/malaise/tiredness, fever, breathing difficulties, decreased appetite, stomach cramps and frequent urination. In Cambodia, because of the seasonal nature of illness at the site and the fact that TME coincided with the rainy season when a high proportion of community members usually suffer minor health complaints, such as common colds and influenza, it was particularly difficult to disentangle actual from perceived side effects. One Cambodian respondent admitted that one person experienced such “side effects” without taking the anti-malarial.

As well as being inconvenient and promoting some health concerns amongst respondents, in Cambodia, adverse events thought to be related to the antimalarial drug administration, here referred to as “side effects” were also seen as having a negative financial impact. In Laos and Cambodia, despite the health care provided by TME staff, people wanted to visit private healthcare providers to obtain intravenous (IV) drips for these complaints. At these sites, IVs were preferred to oral treatment of minor health complaints, because–as described by Cambodian respondents–of their perceived “energising” effect. There were also worries about the opportunity costs of these side effects: people would be too ill to work on their farm or in the forests after taking the drugs.

Despite these complaints, a high proportion of community members participated in the MDA, although they did have an impact on uptake in subsequent rounds. In Cambodia and Laos, study staff responded with specific efforts to minimise the impact of perceived side effects on coverage. Cambodian staff were aware of seasonal disease patterns and sought to explain the perceived side effects in terms of the time of year and seasonal infections. In Laos, TME personnel took several measures to counteract the negative influence of perceived side effects: study physicians spent more time in the villages; the quantity and range of cost-free medicines was increased; and health centre staff together with TME staff visited the households of participants who reported complaints.

Across the sites, there were concerns about the blood collection, which as a component of the experimental TME pilot studies, were used to assess the impact of the intervention on *Plasmodium* prevalence. These were articulated as stories about large quantities of blood being sampled or blood being taken forcibly. In some instances, this had an impact on uptake, with some community members who refused to participate citing blood draws as reasons for non-participation. The people who refused to participate for such reasons were often clusters of friends or relatives. Staff involved in TME were aware of the potential impact of these stories and their spread. When they emerged, targeted efforts were made to understand and rectify underlying concerns. For example, village authority figures were asked to participate in community meetings to address concerns and explain the blood sampling procedure.

### Trust, social relationships and community engagement

At each site, an intensive programme of community engagement activities accompanied the TME study. The primary aim of these activities was to promote uptake of the MDA in the target communities. Besides dealing with the perceived side-effects, study staff faced a host of challenges when seeking to encourage participation. Difficulties, included, in Laos and Cambodia, dealing with the communities’ past experiences of NGOs working in the local area and perceptions that they had not kept their promises. In Myanmar, previous of MDAs against filariasis had left unpleasant memories and prompted concerns that the government was attempting ethnic cleansing.

Determining which elements of community engagement were effective in overcoming barriers and reluctance was complicated by respondents’ difficulties in disentangling community engagement from other study-related activities. For study staff in Myanmar, community engagement was more than a set of community meetings; it was present in all study activities. Community members in Laos also found it difficult to assess their preferred community engagement activities and valued the study as a whole. In Cambodia, participants were often unable to describe community engagement activities separate from drug administrations and surveys, referring to TME as a single undertaking. At all TME sites, engendering trust was viewed by participants and staff as integral to effective community engagement, and critical to achieve adequate coverage.

Trust was seemingly built in a number of ways. Study staff’s participation in local social activities, such as funerals and local festivals, their presence in the villages and commensality–sitting round the same table and sharing traditional food–helped to build trust. Trust was also built in more structured ways, for example through collaborating with authority figures from the national malaria control programmes, provincial governments, district councils, village leadership and health centres. To this end, in Myanmar, study staff involved a range of authority figures in community engagement activities. These included village leaders or other key individuals, such as teachers, village health workers, traditional healers, monks and representatives of armed groups also participated. In Myanmar, hard-copy approvals with official stamps from regional (Kayin/Karen) authorities were also key to overcoming some of the concerns about MDA.

At all sites, local residents were involved in study implementation. The “volunteers”, who usually received participation incentives, were generally selected by the local communities. The local village malaria worker or community health worker were sometimes chosen for this role. They took on specific key roles, informing the community members about the study activities, encouraged participation, took part in MDA, helped to follow-up with community members who missed a dose or round of MDA, and acted as a point of contact for reporting adverse events. This combined with the involvement of authority figures in community engagement, was intended to engender ownership among the residents. In Cambodia, following a drop in coverage in the second round, study staff took further measures to promote ownership: this involved local health staff taking the leading roles in community meetings. Subsequently, coverage increased in the final round of MDA.

Gaining genuine trust in the communities was sometimes a complex undertaking. In Myanmar, “ah narr”, which entails avoiding social embarrassment, observing social niceties or being hospitable, influenced social interactions. For example, showing agreement by nodding, did not always mean that community members understood the explanations or would ultimately participate.

### Local healthcare and economic resources

At all sites, ancillary healthcare was made available to all members of the community, regardless of whether they participated in the MDA or not. In each country, the design of this care was adapted according to the needs of local communities, resources available and local practicalities. In Laos, formative social science research informed the design of the care, and in all sites, consultations with community leaders were important. The care was also adapted to changes that occurred during the duration of TME. The ancillary care proved popular in the target communities, where healthcare facilities were often limited. In Laos, for example, study staff saw overwhelming demand on the mobile health services provided by TME staff. In Myanmar, staff took measures to avoid over treatment in light of excessive demand. In the study settings, it is unsurprising that the ancillary care was popular. Particularly in Myanmar and Laos, target communities were isolated, adequate health facilities were scarce, resulting in limited access to healthcare. Access to healthcare was further impeded by a lack of money that caused difficulties in meeting travel and opportunity (time) costs and paying treatment charges.

Providing essential health assistance demonstrated to villagers that TME staff were concerned about their welfare. This form of engagement fostered confidence in the intervention. Delivering MDA on a house-to-house basis (as was the case in Laos and Cambodia in 2016) was viewed in similar light: participants were able to interact with staff on a one-to-one basis or in small groups to ask questions. Such home visits were considered a gesture of care, demonstrating that staff were attentive to their needs and circumstances. Negotiating the limits of ancillary care however raised challenges to relationships and the confidence that had been built, particularly the high demand for hard-to-justify medicines (e.g. infusions).

At all but one study site, where a community-level incentive alone (such as a water pump for the community) was provided, financial compensation was offered to participants when taking each dose of the antimalarial delivered as MDA. The provision of incentives was determined by the national ethical review boards (ERBs). The amount was determined by study staff, based on an assessment of the value of the labour that local people had to forgo to participate in the MDA and approved by the respective ERBs. In Cambodia, in 2016, the 2^nd^ and 3^rd^ rounds of MDA were delivered without financial compensation. When asked about this, community members reported not paying particular attention to this change and described their motivation for their continued participation in terms of the value for their communities. Contrary to expectations, coverage increased despite no remuneration being offered.

The lack of healthcare resources reflected the poverty and isolation of the target communities. Community members’ poverty played a complex role in influencing uptake of MDA: on the one hand, people were concerned about the economic implications of a bout of malaria; participating in the MDA was thus viewed as an insurance against loss of income. On the other hand, participants were worried about the economic consequences of the perceived side effects that they associated with the anti-malarial drugs.

### Division and unity in isolated target communities

Local community dynamics also played a role in the coverage of TME. In Laos, study staff observed a general conformism in the villages: with community members’ behaviour often influenced by that of other household members and neighbours. Hence, the participation of members of a household in MDA encouraged their neighbours to join in. The household nature of decision-making was observed across the sites: household heads could strongly influence the participation of other household members.

In Myanmar, some village shopkeepers, were located in the settlement and profited from other residents’ purchases but did not consider themselves as part of the community. Community members who worked in Thailand and their relatives were also sometimes wealthier and more educated. This seemingly weakened their sense of belonging to the local community, and appreciation of the possible collective benefit of MDA. This self-imposed exclusion from the village life made it harder for staff to attract these groups to participate in the study. Political allegiances also played a role in Myanmar: in one community, the study was viewed as aligned with one particular political group; people who felt affinity to opposition groups, who comprised around half of the population, refused to participate. Coverage in this village was particularly poor (below 50%).

In Cambodia, the 2016 rounds of TME targeted communities comprising predominantly ethnic minorities. Study staff indicated that community members from the ethnic minorities tended to participate in the MDAs and did not present a barrier to high coverage. They explained this observation, at least in part, in terms of the involvement of village leaders in community engagement: once the leaders agreed, other community members would also participate. The challenges around the participation were often related to the isolated nature of these settlements. Absence of passable roads and poor mobile phone coverage meant that informing villagers of changes to the study routine was often difficult. Such difficulties reflected broader challenges faced by study staff throughout the sites. Poor transport infrastructure meant that accessing communities was sometimes impossible, and when possible, it implied long and difficult journeys. In Myanmar, the intense challenges of the work led to high staff turnover. New staff had to start again in terms of developing social relationships.

## Discussion

Mass antimalarial administration has been proposed as a strategy to eliminate *P*. *falciparum* infections rapidly from remaining transmission foci in the GMS [6, 7]. Recent TME pilots studies have evaluated the effectiveness of a package of interventions, including MDA, to interrupt falciparum malaria transmission, [18–23]. In target communities, high coverage of MDA (typically estimated at above 80%) is essential to maximize its impact on transmission [25]. Using qualitative and quantitative methods across pilot study sites in the GMS, this programme of research explored the factors that influence coverage of MDA for malaria elimination.

Overall MDA coverage was adequate for a single round (>80%) but below the estimated level to interrupt transmission for all three rounds (55%). Variation in participation across sites was influenced by a complex set of factors, with community engagement activities intended to promote coverage, intertwined with the context in which the studies played out. Reasons for participating included a general appreciation of the value of addressing malaria in their communities [22, 23]. Awareness of TME was associated with participation but respondents often had an incomplete understanding of the rationale for MDA. Trust in those providing information about the intervention therefore prompted participation. Other benefits of participation, financial compensation and free-of-charge ancillary care [23], played a role, but the continued high uptake in Cambodia when financial compensation was not offered indicates that the importance of incentives can be overstated [38]. The impact of political and social divisions and cohesion highlights the limits of community engagement in some circumstances.

Familiarity with the causes and symptoms of malaria was associated with participation in MDA. The qualitative data also indicated a general appreciation for efforts to eliminate malaria in the communities: despite general declines in clinical cases across the region [4], malaria was often seen as a health issue. This was particularly the case in Cambodia, where there have been concerted and visible efforts to contain the spread of resistant falciparum strains. This has included the large-scale recruitment and training of village malaria workers (VMW), plus the distribution of effective anti-malarials, rapid diagnostic tests, long-lasting insecticide-treated bed nets (LLINs) and hammock nets, engagement with private drug sellers to address the sale of counterfeit and sub-standard anti-malarials, and enforcing the ban on the sale of anti-malarial monotherapies [43].

Malaria infection was viewed as intertwined with forest activities, particularly in Myanmar. This reflects consensus among malariologists regarding the importance of forest transmission in the GMS, where the main vectors are the exophagic and forest dwelling *A*. *dirus* and *A*. *minimus* [44]. For forest-going members of the target communities, conventional vector control interventions are likely to offer limited protection from malaria. As has been reported elsewhere in the region, insecticide-treated hammocks or ITNs [45–47] are little used or irrelevant because of the nature of forest activities, such as night-time socialising, urinating, defecating [48], logging or hunting [48, 49]. Forest-going is often an integral aspect of local livelihood activities in target areas and therefore places many community members at risk of infection. Concern about suffering a bout of malaria was also linked to its economic impacts because it usually meant a time of unproductive convalescence away from the farm and forest.

The transmission potential of “sub-clinical infections” is a central concept in the rationale for MDA and although this idea resonated with some potential participants (e.g. in Laos and Cambodia), respondents generally referred to symptomatic malaria and the notion proved difficult to grasp even for some study staff [14, 23]. It is therefore likely that community members often participated in TME without fully understanding its rationale. This emphasizes that promoting coverage entailed more than providing a comprehensible explanation of the study.

Attitudes to MDA were influenced by the adverse events that respondents associated with the anti-malarial. At each site, coverage decreased over subsequent rounds, and community members’ experience of the drug influenced their readiness to participate again [14]. In the absence of a placebo control group, it was not possible to disentangle actual from perceived side effects. This was underlined in Cambodia, where MDA in the rainy season coincided with seasonal minor health complaints, such as common colds and influenza. The question of whether adverse events were causally related to participation in the MDA was however irrelevant: people were worried about their potential health and financial impact: seeking treatment–sometimes IV “kits”–from private health providers was expensive; and there might be opportunity costs (absence from work in the fields or forest).

Across Asia, IVs–usually delivering saline and sometimes antibiotic–are a popular remedy for general, non-specific health complaints. Their popularity has been explained in terms of similarities with acupuncture and the widespread use of injections by barefoot doctors in rural China [12, 50–52]. Cambodian respondents described the preference for IVs in terms of their “energising” effect. This–and the overlap between seasonal colds and influenza–highlights how the anti-malarial was viewed in terms of a threat to one’s physical strength, with concerns focused on fatigue and malaise (and their potential impacts on productivity). Elsewhere, anthropologists have emphasized the need to understand how the unwanted effects of anti-malarials are interpreted in terms of wider ideas about well-being and take them into account when advocating adherence [53]. At these sites, community engagement was adjusted to address these concerns, particularly by the more prominent involvement of local stakeholders [14].

In seeking to provide a comprehensible explanation for the study rationale and procedures and to address the impact of perceived side-effects, community engagement incorporated a variety of approaches to disseminate information about MDA. These included banners, posters, community meetings, social media and face-to-face interactions on a one-to-one basis or in small groups. At village level, the activities were intensive and tailored to the local context and audience, with the mix of approaches varying across sites and over time. In Cambodia, for example, community drama proved popular [54, 55]. Interactions at participants’ home were also valued because they provided opportunities for potential participants to quiz the field staff. Home visits were viewed as a gesture of concern [41] and enabled more informal interactions and rapport to be built. In all these activities, simple and locally tailored messages about MDA as a strategy for malaria elimination seemed to resonate in communities and were particularly appealing to participants in settings where malaria was considered a priority health concern [8, 9, 12, 14, 18, 21, 27].

Spending prolonged periods in the target villages helped TME staff to understand local social dynamics, village calendars, and mobility patterns (e.g. time spent in forested areas). This experience combined with formative and on-going research to adapt community engagement and study activities according to the local context and to events that occurred during the study period [21, 27]. For example, in Cambodia, study staff identified influential opinion leaders who did not occupy formal roles in local hierarchies but helped to address outbreaks of perceived side effects during the rainy season.

In all TME sites, community members took active roles in implementing the study. Local authority figures were involved in the recruitment and training of volunteers. This sharing of responsibilities with community members reflects elements of the community-directed approach to research [21] and positively influenced participation, particularly, in Cambodia, where after the second round of MDA, district authorities and local community leaders took over community engagement [16].

Social relationships were key for the participants’ responses to the information provided about TME. Trusted members of the community, such as community health workers and senior community members took up important roles in influencing decisions about participating in MDAs. Convincing influential community members of MDA’s importance was essential in gaining credibility. This entailed the formal engagement of–national, regional, provincial and village–stakeholders particularly in the initial planning stages and gaining approvals in a sequential manner to build confidence in the study [16, 21] As has been observed in other clinical studies [56], through spending time in the target communities and participating in everyday life, MDA staff were able to build relationships that promoted trust in the messages they were offering.

As in other studies [57], faced with the complex rationale for the intervention, participation in MDA was influenced by the trust that community members placed in study staff. Developing social relationships–grounded in the sharing of time, food and resources–was an important component of this trust [9]. Such relationships can however blur the boundaries between intimacy and detachment, participant and researcher [57]. Moreover, in contexts where reciprocity and obligation are social imperatives, such as in the GMS, social pressure and conformity can drive participation [23]. In Laos, social conformity played a notable role in participation: households participated in MDA because they saw others doing. Care is therefore needed to ensure that building trust through social relationships does not detract from providing full and comprehensible information about the study.

The lower levels of coverage at each round in Myanmar I were caused in large part by local political and social divisions in specific target communities. One village comprised two factions that were originally from separate settlements that had coalesced as each had increased in population. The pilot study was viewed as being aligned with one faction and hence participation among members of the other was low. In another community, local social fragmentation had resulted from recent migration to the area and the sensation among recent arrivals–particularly Burmese shopkeepers–of being distinct from other community members. This meant that they did not see the intervention as relevant for them [9]. This highlights the limits of community engagement, regardless of how well it is tailored to local contexts, and underlines the need to avoid simplistic conception of community [58].

Decisions about participation were taken in the background of concerns about the economic impact of a bout of malaria and potential side effects from the MDA anti-malarial. Participation had economic consequences in the form of compensation provided in cash and/or household utensils. However, in Cambodia, when no cash compensation was provided for participation in rounds two and three in 2016 coverage unexpectedly increased. When asked, respondents downplayed the role of the cash reimbursements. Entire villages were able to access the free ancillary care that study staff provided and the infrastructure improvements that were made (e.g. providing a water pump).

### Lessons from pilot studies to implementation

Many of the issues highlighted in the findings of this study were exacerbated by the experimental nature of TME. Mass anti-malarial administration was accompanied by a plethora of additional activities necessary to fulfil study requirements and characterise the effects of the intervention package, including detailed informed consent procedures and periodic blood sampling. As in many settings, if medical research involves blood sampling, concerns often about these procedures and their impacts on the health of participants [59]. In the case of TME, some of these anxieties are likely to have reduced coverage. There are also important but more subtle differences between MDA under experimental and implementation conditions. Clinical trials often engender tacit assumptions of reciprocity whereby participants are aware that they make an essential contribution to the study, through their participation (and occasionally supplying physical samples, such as blood) and hence expect–explicitly or otherwise–something in return [57]. The intensive nature of study activities can also engender concern: emphasizing potential adverse events of the current first-line anti-malarial can prompt worries, as can offering (visible) emergency healthcare [38]. Additional resources to promote coverage, such as offering ancillary care and financial compensation, contributed to overcoming barriers that were associated with TME’s experimental nature.

Even though the challenges related to achieving the necessary level of coverage of TME were exacerbated by its experimental nature, similar issues have been described in the roll-out of MDAs that target infectious diseases other than malaria. As part of the WHO’s Global Programme to Eliminate Lymphatic Filariasis [60], national disease control programmes conduct MDA without incentives to promote participation. In Tanzania, for example, social scientists have highlighted how the uptake of a MDA targeting filariasis was influenced by concerns about medicines (prompted by distrust in overseas organizations), local understandings of lymphatic filariasis, and inadequate communication about the rationale for MDA [61]. Similar findings have also been reported in Uganda, where MDA for schistosomiasis control has been implemented as part of the programme for Integrated Control of Neglected Tropical Diseases [62].

Recent data from the roll-out of MDA for malaria elimination as part of the Malaria Elimination Taskforce in Myanmar indicate that under non-experimental conditions coverage was higher than for the pilot study in the same area: in the target villages at scale-up, over 90% of community members participated in one round or more of MDA (median 91% IQR 86–95, n = 50 villages), almost two-thirds participated in three rounds (64% IQR50–78) [63]. The programme of community engagement undertaken by the Malaria Elimination Taskforce also emphasized two key elements identified in this programme of research: the crucial role played by trust and social relationships, and the need to invest time and effort in understanding the everyday lives of members of target communities [64]. The taskforce’s success was also embedded in a broader strengthening of local primary care, which has been recognized as key to the potential for successful malaria elimination in areas where healthcare infrastructure is often limited, as is generally the case in areas of the GMS where malaria transmission continues [65].

### Recommendations for the potential scale-up of mass antimalarial administration

- All aspects of community engagement must be tailored to local (social, cultural and political) circumstances and must be responsive to events and feedback during the intervention period
- Tailored and responsive community engagement requires an understanding of target communities, which can be garnered through formative and ongoing research, and through involving community members as part of the intervention team
- Clear, simple and locally tailored messages about MDA for malaria elimination are an important element of community engagement but to maximize the impact of these messages, positive social relationships must be engendered between community and intervention team members
- To build the necessary trust, those implementing the programmes must invest considerable time in communities and be prepared to interact with community members appropriately, demonstrating respect for their customs and opinions
- Considering the challenges of community engagement in geographically isolated areas, intervention team members require careful training and intensive support
- Any tendency to interpret MDA as prophylaxis, and neglect other malaria prevention and control practices, such as ITN use and treatment seeking, must be addressed. Carefully tailored messages play an important role in achieving this

### Strengths and limitations

This research combined a range of qualitative data collection techniques with questionnaire-based surveys and recruited various respondent types from a range of contexts. It is thus the largest combined analysis of qualitative and quantitative data on factors affecting the uptake of mass anti-malarial administration to date. The mixed-methods approach enabled patterns in factors across the various sites to be identified and explored in-depth. Employing a variety of qualitative data collection techniques enabled the triangulation of findings and reduced the undue impact of a particular method. To reduce to potential for bias to be introduced by a specific individual, at each site, data were collected by a team of researchers, who were debriefed regularly by a senior social scientist and other senior members of the study team. Site-specific efforts to reduce the potential for bias included gender-specific FGDs in Laos to ensure that women’s perspectives were incorporated in context where there is a general deference to men when in mixed groups.

The data were collected alongside a cluster randomized trial, which entails a range of research activities besides MDA. These circumstances bear little resemblance to a potential implementation of MDA for malaria elimination as part of public health policy, which would generally be rolled out without research-related procedures. There are therefore limits to the recommendations that can be extracted from these findings for scaled up MDA as a tool for malaria elimination. Furthermore, for practical reasons, not all data collection techniques could be employed at all sites, with qualitative data absent from Vietnam.

## Conclusion

Across study sites in four countries of the GMS, a variety of issues affected the coverage of MDA for malaria elimination: understandings of malaria and MDA; the perceived positive and negative consequences of antimalarials; trust and social relationships that were fostered, in part, by the community engagement activities; local economic and healthcare resources; and divisions and conformity in target communities. The experimental nature of the pilot studies presented particular challenges to the achievement of high coverage, for example, concerns about blood draws negatively affected participation. Nonetheless, the findings resonate with those from social science research of MDA under implementation conditions (e.g. for filariasis) and offer useful guidance for the design of future roll-out of MDA for malaria elimination in the GMS. Key lessons include the need to understand target communities and to use these insights to provide appropriate information in suitable ways, using an approach to community engagement that recognizes the importance of trust and social relationships.

## Supporting information

S1 Table Logistic regression model for factors independently associated with complete participation in the MDAs. (DOCX)
