# Supplementary material for: Community engagement, social context and coverage of mass anti-malarial administration: Comparative findings from multi-site research in the Greater Mekong sub-Region
Source: PLoS One. 2019 Mar 25;14(3):e0214280. doi: 10.1371/journal.pone.0214280 (PMC6433231; doi:10.1371/journal.pone.0214280)
Supplement: S1 Table — (DOCX) [file pone.0214280.s001.docx]

**Table S1** Logistic regression model for factors independently associated with complete participation in the MDAs

| **Characteristics** | **Participation in MDA** | | | | | |  |
| --- | --- | --- | --- | --- | --- | --- | --- |
|  | **Yes** | **No** | **Complete** | **Univariate analysis** | **p value** | **Multivariate analysis** | **p value** |
|  | **Number (%)** | **Number (%)** | **Number (%)** | **Crude Odds Ratio (95% CI)** | | **AOR* (95% CI)** | |
| Malaria is transmitted by mosquitoes | 703 (83.7) | 137 (16.3) | 414 (89.6) | 2.65 (1.81 to 3.89) | <0.001 | 2.24 (1.42 to 3.52) | **0.001** |
| Symptom of malaria is fever | 507 (60.4) | 331 (39.4) | 313 (67.7) | 1.97 (1.48 to 2.61) | <0.001 | 2.0 (1.42 to 2.83) | **<0.001** |
| I have heard of MDA | 768 (91.4) | 72 (8.6) | 451 (97.6) | 7.89 (4.08 to 15.23) | <0.001 | 5.81 (1.91 to 17.66) | **0.002** |
| I heard about MDA during community engagement activities | 582 (71.1) | 237 (28.9) | 380 (83.5) | 4.06 (2.94 to 5.61) | <0.001 | 2.99 (1.84 to 4.86) | **<0.001** |
| It is important to take medicine | 731 (87) | 109 (13) | 416 (91.4) | 1.8 (1.2 to 2.71) | 0.004 | 1.52 (0.89 to 2.59) | 0.12 |
| I have received enough information | 454 (54) | 386 (46) | 274 (87.8) | 1.6 (1.21 to 2.1) | 0.001 | 1.04 (0.67 to 1.62) | 0.83 |
| MDA medicine is to protect from malaria | 519 (76.8) | 157 (23.2) | 318 (83.5) | 2.36 (1.63 to 3.4) | <0.001 | 1.12 (0.72 to 1.75) | 0.6 |
| Would take medicine again (next year) | 525 (62.5) | 315 (37.5) | 292 (94.2) | 1.06 (0.8 to 1.41) | 0.64 | 0.63 (0.39 to 1.02) | 0.06 |
| *Adjusted for socio-demographic variables | | | | | | | |
